# The TB vaccine development pathway – An innovative approach to accelerating global TB vaccine development

**DOI:** 10.1016/j.tube.2020.102040

**Published:** 2021-01

**Authors:** Danielle Roordink, Ann Williams, Bernard Fritzell, Dominick J. Laddy, Emmanuelle Gerdil, Anne Marie Graffin, Dereck Tait, Leo van der Pol, Ilona van den Brink, Marit Holleman, Jelle Thole, Gerald Voss, Maria Lempicki, Georges Thiry

**Affiliations:** a TuBerculosis Vaccine Initiative (TBVI), Lelystad, the Netherlands; b IAVI, New York, USA; c IAVI, Cape Town, South Africa

**Keywords:** Tuberculosis, Vaccine, Research and development, Stage gates, Pipeline, Rational selection

## Abstract

Two proof of concept clinical trials with TB vaccines demonstrate that new approaches can prevent sustained TB infection in adolescents (BCG revaccination) and TB disease in adults (M72/ASO1_E_) (Nemes et al., 2018; Tait et al., 2019) [1,2]. Both approaches are in late stage development and provide motivation and rationale to invest into a global TB vaccine pipeline. This pipeline needs to be diverse to address TB-specific challenges including variation in target populations, uncertainties in animal model predictivity and lack of immune correlates of protection. It requires that individual vaccine candidates must be advanced rationally and that the global pipeline must be managed in the most nimble and resource-efficient way, especially in the current constrained funding environment. The TB Vaccine Development Pathway is a webtool which has been developed as an offer to the field to provide a source of information and guidance covering vaccine development from discovery to implementation. It is underpinned by generic and TB vaccine-specific guidelines, regulatory frameworks and best practice, and was compiled by a multi-disciplinary team of scientific and technical experts with the input of the TB vaccine community. The Pathway is a unique tool to guide and accelerate the development of TB vaccine candidates and may be useful for other vaccine development fields.

## 1 Introduction

*Mycobacterium tuberculosis* (Mtb), the causative agent of tuberculosis (TB) kills more people than any other single pathogen and remains one of the biggest global health burdens, with ten million new TB cases each year and 1.2 million TB-related deaths in 2019. It is estimated that approximately 23% of the world's population (1.7 billion people) have latent TB infection (LTBI) and that 10% of people with LTBI will eventually develop TB disease [3]. Also, drug resistant Mtb is a major contributor to the burden of antimicrobial-resistant (AMR) infections, which are a major threat to global health security. The disease is complex, with many variables (age groups, geographic or socio-economic factors, co-morbidities) all of which have an impact on strategies for disease control. The current vaccination strategy for BCG, given at birth is not sufficiently effective at reducing disease burden in all target populations and new, improved vaccine approaches are urgently needed. The WHO End TB Strategy sets ambitious targets for reduction of disease burden and in 2018 the United Nation's High-Level Meeting on TB declared a commitment to fight TB. However, disease impact and health–economic modelling has clearly demonstrated that without the availability of new TB vaccines the WHO targets will not be met using current approaches to TB-control [4]*.* Without new TB vaccines the substantial economic burden of TB will continue, with an estimate annual global cost of $10.1 billion in 2019 for diagnosis, treatment and prevention and $12 billion in lost productivity and wages [5]. To ensure that novel, effective and affordable vaccines against TB become a reality, it is essential to sustain a diverse vaccine development pipeline. TB R&D funding is constrained compared to other diseases (for example HIV) [6] and therefore it is critical to rationally progress or discard candidate TB vaccines early in the development process in order to concentrate scarce resources on those candidates with a high probability of success.

Vaccine development is a data-driven, complex and costly endeavour that can take as much as 10–15 years from discovery to implementation, although accelerated development is feasible with sufficient resourcing and political will. This is evidenced by the rapid implementation of Ebola vaccines and the current situation with COVID-19 vaccines some of which are on a trajectory for implementation within a year of discovery. Vaccine development involves multiple decision and investment points, with inherent risks which must be managed. Compared to other diseases, the development of vaccines against TB is even more challenging due to the complexities of the disease, its pathogenesis, the absence of a confirmed hypothesis on immune-mediated protective mechanisms and the relatively lengthy timeframes required for efficacy endpoint evaluation in the clinic. A key challenge in TB vaccine development is the lack of accepted or validated immune correlates or surrogates of protection. This has an impact on preclinical screening of potential candidates and on optimisation of the vaccine formulation, since lengthy and costly protection experiments must be performed using animal models that have not yet been validated to predict clinical efficacy. The lack of an established immune correlate also hampers the development of an appropriate, qualified assay to measure potency which would accelerate and harmonise product characterisation and Quality Control. In response to a need to address gaps in available product development information relating specifically to TB and access to expertise in vaccine development and to provide a more co-ordinated and harmonised approach, experts from IAVI (formerly Aeras) and the Tuberculosis Vaccine Initiative (TBVI) developed guidance for development of TB vaccines. They followed a stage-gating process commonly used in project management [8] where activities of large and complex projects are grouped into stages representing distinct periods of the development process. Movement through the stages is controlled by means of structured evaluation against pre-defined criteria (the gates). This framework is designed to properly plan discrete sets of activities, to monitor actual progress along each specific stage and to allow objective decision-making whether a vaccine candidate can rightfully enter a next stage. It is also a method to highlight and manage uncertainties and minimise risk. In 2012, Barker et al. described progressive development stages pertinent to TB vaccines, proposed the establishment of gates at several critical stages and the desired characteristics of the vaccine at each stage, which formed the criteria to be met in order to pass from one stage to the next [7]. The 2012 publication was the starting point of the TB Vaccine Development Pathway initiative, which aimed to re-evaluate the stage gate criteria in the light of new information and lessons learned e.g. from the results of product development, preclinical and clinical trials and to revise or expand as necessary.

It is important to note that during the development of the Pathway, promising results in both preclinical and clinical development were published. A Cytomegalovirus (CMV) vectored TB vaccine and the intravenous (IV) administration of BCG [9,10] to NHPs both demonstrated significant efficacy in preventing TB disease. M72/AS01_E_ was the first candidate TB vaccine to demonstrate efficacy in preventing TB disease in individuals with LTBI and BCG administered to adolescents with no evidence of LTBI was efficacious in preventing sustained Mtb infection [1,2]. These ground-breaking results placed the TB vaccine field at a significant inflection point as they support the feasibility for the development of new TB vaccines and will also potentially identify critically important correlates of protection. These encouraging results provide a strong scientific rationale to further develop and maintain a pipeline of investigational vaccines with multiple vaccine approaches in order to cover the wide range of needs. In order to sustain this pipeline, balanced investment is needed for which rational tools for individual candidate assessment and progression as well as for portfolio decision making, are important, particularly to ensure efficient use of limited resources [[11], [12], [13]]*.* The overall objective of the TB Vaccine Development Pathway is to develop such methods and guidance for TB vaccine-specific challenges. The Pathway is an offer to the field, does not pretend to be prescriptive and may need to be adapted or rendered more granular for individual vaccine development projects.

## 2 Materials and methods

The motivation for the TB Vaccine Development Pathway tool was to re-evaluate and refresh the existing stage-gate criteria published in 2012 [7], to add further stages covering activities from discovery to launch and to include explanatory guidance notes. The aim was to incorporate contemporary expertise, best practice and lessons learned in TB vaccine development and advances in the TB field alongside generic guidelines, for example for technology platforms or regulatory compliance. Whilst not aiming to be prescriptive for each vaccine project, a key objective was to address specific TB vaccine target populations or indications for which there may be different types of vaccines or distinct approaches.

### 2.1 The TB vaccine development pathway working group

**Fig. 1:**
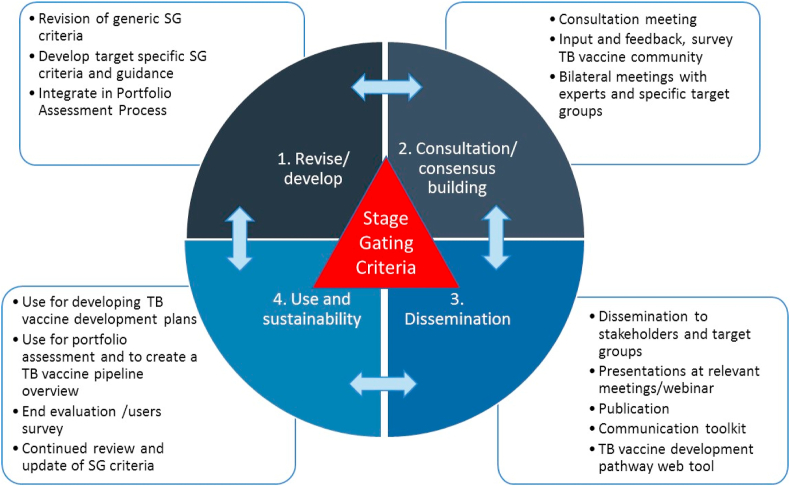
Schematic illustration of the various steps involved in preparation of the Pathway. The process consisted of four working areas (1) revise the stage gate and related criteria, (2) consult experts and users, (3) disseminate to target groups, (4) use of the tool and updates.

The Pathway was developed with contributions from a multi-disciplinary team gathered in a Working Group comprised of all the authors of this publication who each brought scientific and technical expertise in the various aspects or functions of vaccine development, as well as input from the TB vaccine community. The project was initiated in 2017 by the Global TB Vaccine Partnership (GTBVP), an alliance of organisations that aim at making novel TB vaccines a reality, and was funded by the Bill & Melinda Gates Foundation. This overall process of the project to develop and refine the Pathway is illustrated in Fig. 1.

### 2.2 General principles of the design and content of the pathway

The starting point for the Pathway project was the 2012 publication by Barker et al., describing a “Rational approach to selection and clinical development of TB vaccine candidates.” where the activities and success criteria for each stage were presented in the form of tables. A key objective was to generate a tool which could integrate all the information in a single source that would be useful across all of the functions and stages of development, from discovery to implementation. Using the stages as defined in the 2012 Barker et al. publication the Working Group first selected the most appropriate product development functions relevant to each of these stages. The activities included in each stage were defined by sub-groups with expertise relevant to the specific function. Finally, the criteria needed to consider a stage completed and support the progression of a project from one stage to the next were defined. In addition, guidance notes were generated to provide supporting information as well as references specific to each function within each stage.

### 2.3 Sources of information

Although there are many sources of guidance relating to vaccine development there is limited publicly available information which specifically describes the requirements for development of a new TB vaccine. There are many factors that contribute to the exceptional nature of TB vaccine development, whether preclinical, clinical or manufacturing. Whilst there may be some aspects e.g. technology platforms such as vectors or subunits for which there is appropriate guidance or precedent from other vaccine fields, few precedents and principles that apply from other vaccine development pathways can be directly transposed to TB vaccines. Of particular note is that for many vaccines, antibodies are a key adaptive immune response and are accepted surrogates for efficacy; these can be quantified using widely accepted, standardised techniques, enabling benchmarking throughout the development stages. In TB there is no clear role for antibodies and no generally accepted alternative immune assay to facilitate standardised comparisons between and within vaccine projects.

A variety of sources of information were used to develop the Pathway. These included TB vaccine-specific literature such as scientific publications [[14], [15], [16]] and the WHO Preferred Product Characteristics [17], as well as publications describing lessons learned from other vaccine fields [18]. A key source of information which underpins large sections of the Pathway is regulatory guidance (WHO prequalification and the WHO Preferred Product Characteristics for TB vaccines) and requirements (for example the ICH-E2C (R2) Guidelines) that are either generic for vaccines or specifically relate to BCG, the only licenced TB vaccine.

### 2.4 Consultation and dissemination process

Consultation and consensus-building is an important consideration in order to generate widely accepted criteria and therefore several steps for consultation and feedback with the TB vaccine community were made. This included an initial survey among the TB vaccine community to obtain general feedback on the appreciation and use of the original Stage Gate (SG) criteria developed in 2012, to gather suggestions for improvement and to identify the needs of the community prior to starting the revision of the SG criteria. Twenty seven responses to the survey were received including responses from key TB vaccine researchers, biotech companies and stakeholders such as WHO, NIH, BMGF and EDCTP. Following the completion of the first draft of the revised Stage Gate tables (describing activities and criteria for each stage) a consultation was held with a broad group of stakeholders which included academia, industry, funders and regulators. The aim was to review the utility of the content (too little or too much information) and to ensure that the breadth of the expertise of the working group was sufficient. Written feedback was provided and a consultation meeting was convened to allow further discussion and recommendations. The meeting was attended by 20 participants, consisting of representatives of TB vaccine research institutions such as Oxford University, Max Planck Institute for Infection Biology, Statens Serum Institute and vaccine development organisations such as AERAS (now IAVI) and biotech and pharmaceutical companies including Biofabri and Transgene as well as the stakeholders mentioned above. Throughout the project, presentations have been made at relevant meetings and conferences which describe the current status and future plans and any comments or feedback were followed up with bi-lateral discussions. A brochure and other publicity materials have been distributed at relevant meetings and conferences. A second survey requesting feedback on the webtool was completed in 2020.

### 2.5 Webtool development

The aim of the design of the webtool was that it would be a publicly accessible and integrative easy-to-use tool that would meet the needs of users wanting to access specific information while providing a generic overview of the development process. The tool was therefore designed using the format of the stage gate criteria tables, based upon those described in Barker et al., 2012 and was built in a way that allowed access to information via different routes. For example, a user interested only in one stage of development would be able to go to a single page showing all the functions (activities) related to that stage while user interested in a specific function would be able to navigate to a page listing the details of that function for all of the stages that apply. Guidance notes may be accessed within each individual table and also contain links to references and other resources.

The stage gate tables were implemented first in the webtool in September 2018 followed later with the additional guidance layer in September 2019.

## 3 Results

### 3.1 Overall view of the TB vaccine development pathway

**Fig. 2:**
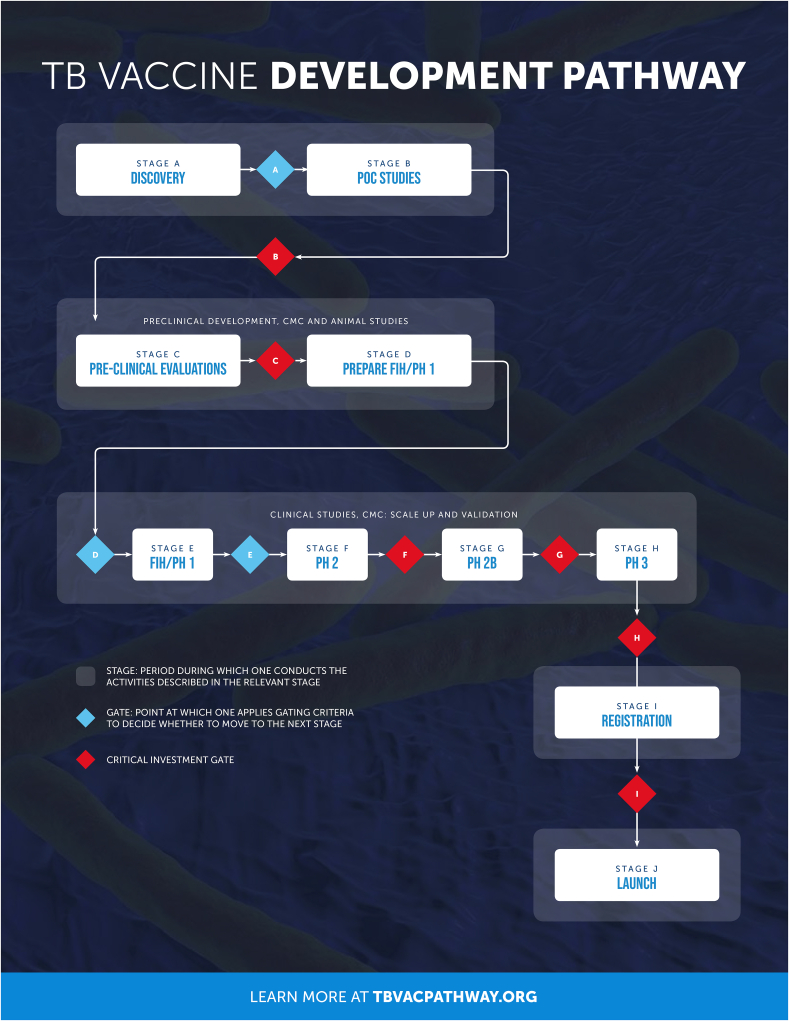
Overall view of the stages and gates of the Pathway (www.tbvacpathway.com).

**Fig. 3:**
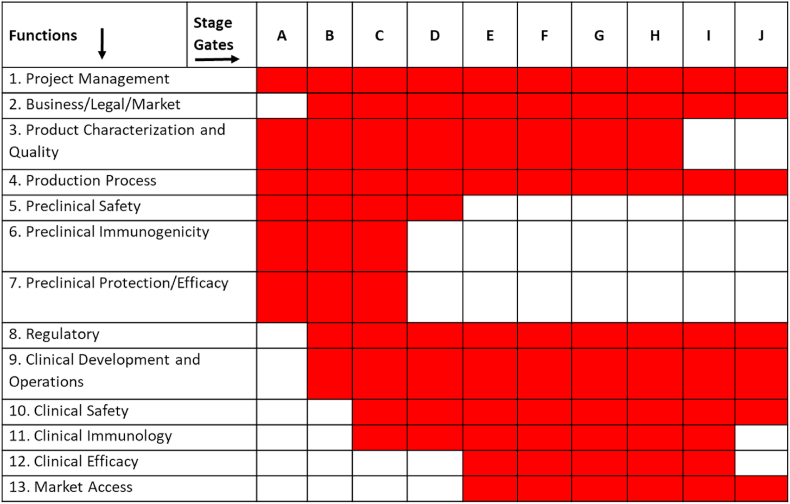
List of 13 functions and the relevant Stage Gates. The Table shows the expertise (function) needed at each stage, and how new expertise are added as the vaccine candidate progresses through the Stage Gates.

The Pathway is comprised of 10 stages from Discovery (Stage A) through to Implementation, (Stage J). These stages are shown in Fig. 2, which indicates the gates that represent the decision point to move from one stage to the next and shows (in red) the gates where a critical or significant investment decision would be made. Stages A and B cover activities in early discovery, stages C and D are relevant to preclinical development, CMC and animal studies (up to and including preparation for first-in-human studies). Stages E though to H describe clinical studies and CMC with scale up and validation. Stages I and J cover activities for registration and launch. The activities to be performed along the development pathway were grouped into thirteen different functions which are listed in Fig. 3. The functions were only developed as relevant to the specific stages, for example the preclinical safety, immunogenicity and efficacy functions were considered only in Stages A through to D.

### 3.2 Tables and guidance

**Fig. 4:**
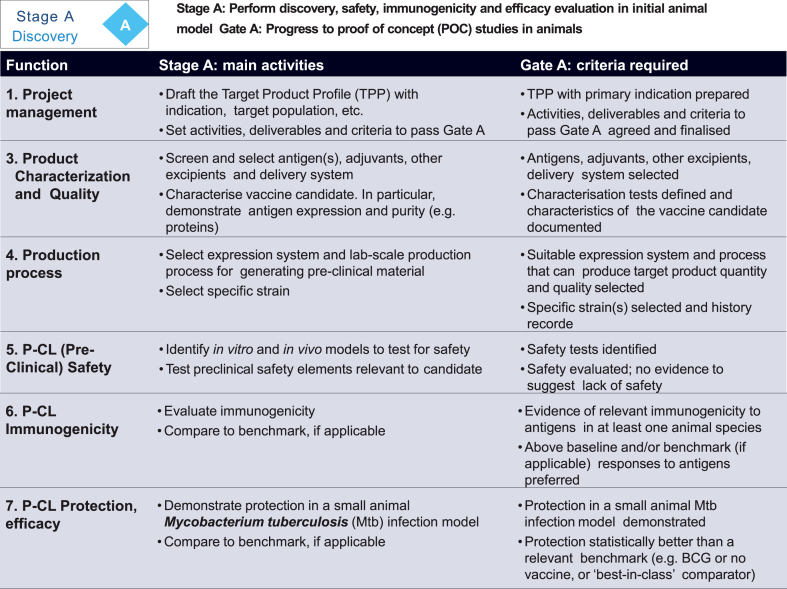
Illustration of stage gate table.

An illustration of how the tables were built is shown in Fig. 4. It lists the functions, on the left, that are relevant to the stage, Stage A Discovery in this example, with the activities listed in the middle and, on the right, the criteria pertinent to each activity. As described above, the content of the tables was created by sub-groups of the Working Group. Periodically, the entire Working Group met to review progress and to cross-check between functions that the type of activity and stringency of criteria were consistent at each stage. The first output was thus a series of activity/criteria tables for each stage, similar to those presented by Barker et al. A revision and refinement of the activities and criteria was made in response to the first stakeholder consultation as in Fig. 1. For the purposes of clarity and ease of use, the amount of information listed in the tables was kept to a minimum and therefore, once the content of the tables had been finalized, each sub-group generated a guidance document which described, for each function, the overall purpose of the activities within that function and how these differ as the product moves from stage to stage. This guidance document also served as a place to provide references and links to additional relevant published guidance and sources of data that underpin the recommendations.

### 3.3 The TB vaccine development pathway webtool

**Fig. 5:**
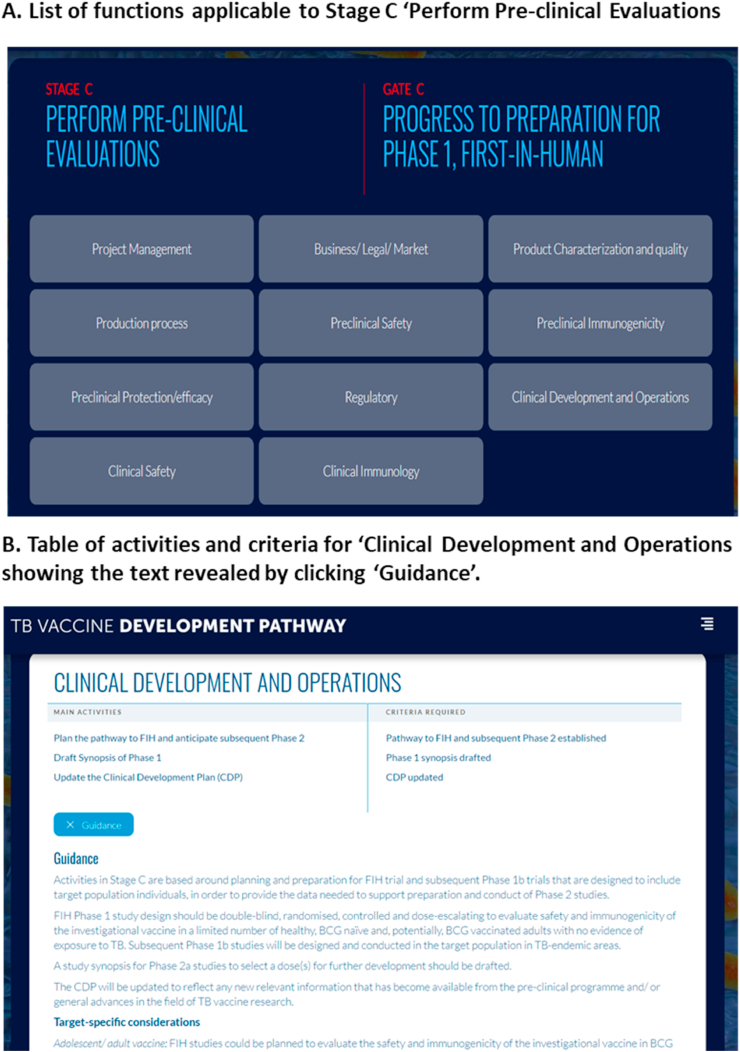
(**a**) List of functions applicable to Stage C ‘Perform Preclinical Evaluations. (**b**) Table of activities and criteria for ‘Clinical Development and Operations showing the text revealed by clicking ‘Guidance’.

The webtool was launched in September 2018, www.tbvacpathway.org. The starting point for the Pathway is the screen shown in Fig. 2. Fig. 5A illustrates the page listing all of the functions relevant to particular stage and Fig. 5B shows an example of the table of activities and criteria and guidance information relating to a single function.

### 3.4 Iterative improvement of the pathway

A revision of the Pathway was made in response to feedback received following consultation, initial dissemination activities and a subsequent survey. The purpose of this revision was two-fold:

1. To develop guidance, to be added to all relevant stages, specific for target populations or indications (namely, neonatal, adolescents/adults and therapeutic vaccines).
2. To add two new stages (registration and launch) such that the Pathway would cover the whole development pipeline from Discovery to Implementation.

The revised online version of the Pathway was launched in September 2019. It is a key objective of the working group that the Pathway is a source of information that is as relevant and current as possible. Therefore, biennial revisions of the content on the webtool are planned to ensure that the latest knowledge and developments relevant for the acceleration of TB vaccines in the global pipeline are included (Fig. 1). Such information would include technological developments such as predictive biomarkers or animal models, new or updated regulatory guidance or clinical trial results that validateor refute previously held assumptions. Revisions will be guided by feedback following consultation and dissemination activities, and will also consider lessons learnt from the development of other vaccines. The next revision of the Pathway will be completed in 2021 and will be conducted by the Working Group based on feedback from represenatives and stakeholders from the TB vaccine field as described in 2.1 and 2.4 and illustrated in Fig. 1.

## 4 Discussion and conclusion

The Pathway is a body of knowledge that offers a data-driven methodology to guide the development of a TB vaccine. It is an offering, made to developers, funders and decision-makers and is not intended to represent a compulsory set of rules although it is aligned with regulatory frameworks and the WHO Preferred Product Characteristics [17]. The objective was to create a source of information that was as TB-specific as possible whilst paying due attention to general considerations and requirements for vaccine development such as criteria taken from GLP, GMP and GCP guidelines. The 2012, Barker et al. publication provided the starting point with the intention to update the information based upon new TB-specific data (scientific publications, human efficacy trial data, guidelines from working groups etc), to extend the scope of the development pathway and to create an interactive web-based methodology for presentation of the information. This second version of the TB vaccine stage gate criteria was created in order (a) to provide even greater specificity for TB by adding guidance relevant to specific vaccine target populations and indications, and (b) add stage gates in early development (from Discovery to Preclinical) and in late stage, down to launch, for granularity and completeness.

The webtool as a platform for the Pathway was developed in order to achieve maximum dissemination to all interested parties and to provide a mechanism for frequent updates and interactions with the users via feedback. Thus, the Pathway is a living set of recommendations and guidance which is underpinned by the status of the TB vaccine field and it is expected that the activities, criteria and benchmarks will evolve as more data become available and advancements are made. This opportunity for continual refinement is an advantage of the webtool as opposed to a publication which is a snapshot in time based upon current thinking and situation. However, a challenge for the webtool platform is providing sufficient information in a format that remains user-friendly. Links to additional sources of guidance provide a mechanism for incorporating more detailed information but there is nevertheless a need to present the key facts in a concise manner. This necessitates the use of terminology that may be unfamiliar to those outside of the TB vaccine field and so a limitation may be that this tool is less useful for those users. However, the webtool is a source of generic information about the principles and requirements for vaccine development, consolidated into a simple to use platform which could be useful to developers of vaccines for other disease areas. Furthermore, by using the same methodology and generic information embedded in the TB vaccine pathway, additional disease-specific vaccine development pathways could be created. It is hoped that the Pathway tool will be multi-functional, having utility for users across disciplines and development stages. For the early stage researcher, the tool can be educational and guide research activities towards product development. For the novice developer the tool can assist in the understanding of the activities and requirements at different stages of development and help them to build robust product development plans. Funders and investors may wish to use the criteria to objectively compare different vaccine candidates in order to assist with financial decision making and more efficient use of resources. For larger and more experienced developers, there is an opportunity to use the community engagement and feedback mechanisms to offer their expertise and experiences in order to enhance the guidance or sources of information in the tool.

The intention is that the Pathway provides a framework such that stakeholders from across the spectrum of TB vaccine development and implementation can gain access to the same information. It is a means of harmonizing different sources of data and to provide consistency. This is particularly important as a means of building confidence between developers and funders/investors as a vaccine cannot advance to the next phase without financial backing. However, to achieve this confidence there needs to be community consensus and acceptance of the content of the Pathway. Thus, the dissemination activities, mechanisms of feedback and iterative improvement are a critical aspect of the Pathway development to date and moving forward. Open data and know-how sharing are encouraged and valued within the TB vaccine community and this open environment has contributed to the development and implementation of the Pathway. It is hoped that common usage of the Pathway and feedback from developers will further perpetuate the open sharing of data and best practice. Foundations and small/mid-size industry who may have fewer resources available to advance candidates may find the Pathway particularly helpful. It offers guidance for construction of development plans and for making critical decisions in moving a single candidate through the pipeline, or in the case of organisations with multiple candidates as a mechanism to support portfolio management.

The development proposed is sequential; stage gate decisions are data-driven and provide a rational basis for careful investment in the context of limited funding. However, as demonstrated by Ebola and particularly COVID-19 vaccine development, this process can be significantly accelerated, with activities run at risk and in parallel, if supported by unprecedented mobilised capacity and investment. Lessons must be learned from Ebola, and soon hopefully from COVID-19, for accelerated development, scale and access and, as appropriate, incorporated into the TB vaccine development stage gate criteria.

The Pathway is a body of knowledge and data-driven methodology to guide the development of a TB vaccine from discovery to commercialisation. It is free to use, interactive and iterative and an offering for developers, funders and decision-makers providing guidance that is aligned to regulatory specifications and the WHO Preferred Product Characteristics. The Pathway offers a structure and guidance for preparing a Target Product Profile and a Product Development Plan for a candidate vaccine, for a team to organize and coordinate activities and operations, and is a tool to make portfolio decisions. It is a unique tool that has been developed to support individual TB vaccine development and to address the need to have a healthy pipeline of new TB vaccines and for targeted, rational and balanced investment.

## Funding

The development of the TB vaccine pathway received external funding from the 10.13039/100000865BMGF, grant number OPP1169968 and title ‘TBVI stage gate design efforts’.

## Author contributions

Ann Williams, Danielle Roordink - writing original draft and editing. All other Authors – reviewing and editing. All authors have contributed to the conceptualization, design and execution of the online tool ‘TB Vaccine Development Pathway’. All have read and agreed to the published manuscript.

## Declaration of competing interest

None.
